# Causal associations between gastroesophageal reflux disease and lung cancer risk: A Mendelian randomization study

**DOI:** 10.1002/cam4.5498

**Published:** 2022-12-08

**Authors:** Lin Li, Qiaoya Ren, Qian Zheng, Yuju Bai, Sisi He, Yu Zhang, Hu Ma

**Affiliations:** ^1^ Department of Oncology The Second Affiliated Hospital of Zunyi Medical University Zunyi China

**Keywords:** gastroesophageal reflux disease, lung adenocarcinoma, lung cancer, lung squamous cell carcinoma, Mendelian randomization

## Abstract

**Background:**

Observational epidemiological studies suggest that lung cancer risk may be raised by gastroesophageal reflux disease (GERD); however, the causal relationship between them remains unknown. Our study performed the two‐sample Mendelian randomization (MR) approach to examine the causal relationship between GERD and lung cancer.

**Methods:**

Instrument variables were found to be independent single nucleotide polymorphisms (SNPs) that were highly linked with GERD (*n* = 129,080). Summary data from genome‐wide association studies (GWAS) data were used to determine outcomes for lung cancer (*n* = 11,348), squamous cell lung cancer (LUSC), and lung adenocarcinoma (LUAD). In this study, three MR statistical techniques (inverse variance weighted (IVW), MR‐Egger, and weighted median) were used to examine the potential causative relationship between GERD and the risk of lung cancer. Cochran's Q test, MR‐Egger intercept test, leave‐one‐out analysis, and the funnel plot were all used in sensitivity analyses.

**Results:**

The main IVW method revealed that GERD substantially increased the risk of lung cancer [odds ratio (OR) = 1.37; 95% CI 1.16–1.63, *p* = 0. 0003], which was also supported by weighted median and MR‐Egger analyses. Using IVW estimate, similar causal relationships were also observed between GERD and LUSC (OR = 1.56; 95% CI 1.26–1.93, *p* = 5.35 × 10^−5^) and LUAD (OR = 1.45; 95% CI 1.09–1.93, *p* = 0.01). Although potential heterogeneity was observed in some studies, random effect IVW was not violated by the heterogeneity, indicating that the causal effect was robust.

**Conclusion:**

GERD was positively associated with the risk of lung cancer, for LUSC and LUAD. This study shed light on a new direction for prevent strategy of lung cancer and therapeutic perspectives in patients with GERD.

## Graphical abstract

Overview of the current Mendelian randomization(MR) study. GERD, Gastroesophageal reflux disease. SNP, single nucleotide polymorphism; LD, linkage disequilibrium. IVW, Inverse Variance Weighted; WM, weighted median; ILCCO, Lung Cancer Consortium.

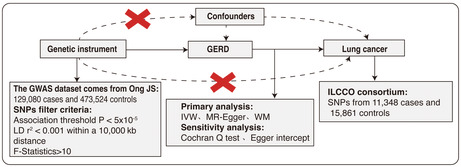

## 1 INTRODUCTION

With 11.4% of all newly diagnosed cancer cases and 18.0% of all cancer‐related deaths worldwide in 2020, lung cancer continues to be the most lethal cancer and the second frequently diagnosed malignancy.^1^, ^2^ Therefore, energetic prevention, early diagnosis, and early treatment of lung cancer are essential. It is also important to identify potentially risk variables since it might help physicians to find early‐stage lung cancer. Although it is commonly acknowledged that cigarette smoking may cause lung cancer, there is currently inadequate evidence to determine the other probable causes of lung cancer.^3^, ^4^, ^5^

Gastroesophageal reflux disease (GERD), a persistent condition in which stomach contents flow backward into the esophagus or trachea, is linked to a number of diseases, including chronic obstructive pulmonary disease (COPD), asthma, idiopathic pulmonary fibrosis, and so on.^6^, ^7^, ^8^ Diagnostic tests for GERD patients usually include upper GI endoscopy and esophageal impedance pH testing. Growing studies indicate that GERD may contribute to lung cancer development, invasion, and metastasis.^9^, ^10^, ^11^ In addition, several observational studies also add evidence that GERD might increase the risk of lung cancer.^12^, ^13^, ^14^, ^15^ Whereas, given the absence of clear evidence from randomized controlled trials (RCTs) between GERD and lung cancer, there is no clear evidence that GERD poses a causal link to lung cancer.

Although RCTs are the gold standard for determining the causal relationship between exposure and outcome, bias can occur as a result of confounding factors and reverse causality. Meanwhile, the procedure of conducting a RCT is time‐consuming, costly, and occasionally impractical as well as unethical. An approach to address this limitation is Mendelian randomization (MR), a method for evaluating causality that employs genetic instrumental factors to proxy for exposures that might otherwise be confounded or prone to reverse causation.^16^ Genetic variants are used as instrumental variable (IV) in MR to investigate the relationship between environmental exposure (GERD) and outcome (lung cancer).^17^ For instance, previous MR analyses have indicated that several factors, such as cannabis use, pulmonary function, rheumatoid arthritis, and multiple sclerosis, are causally associated with lung cancer.^18^, ^19^, ^20^ Unfortunately, the causal link between GERD and lung cancer remained unresolved.

By combining data from published genome‐wide association studies (GWAS), this study first investigated the potential causality between GERD and lung cancer risk through MR analysis.

## 2 MATERIALS AND METHODS

### 2.1 Data sources

Using summary statistics from GWAS, we conducted a two‐sample MR analysis to investigate the causal relationship between GERD and lung cancer. There are three assumptions that have to be met in this MR design: (a) genetic instruments predict the exposure of interest (*p* < 5 × 10^−8^); (b) genetic instruments are not associated with potential confounders; and (c) genetic instruments affects the outcome only through the risk factors.^16^

The primary analysis data were obtained from International Lung Cancer Consortium (ILCCO) (https://ilcco.iarc.fr/) and two large publicly available GWAS abstracts from Jue‐Sheng Ong.^21^, ^22^ This study used GWAS pooled data from Integrative Epidemiology Unit (IEU) public availability (https://gwas.mrcieu.ac.uk/) without requesting through the IEU platform. Specifically, the genetic tool for exposure in this study was derived from recent GWAS pooled data on GERD. A total of 129,080 GERD patients and 473,524 controls were analyzed, and 80 single nucleotide polymorphisms (SNPs) were significantly linked to GERD. ILCCO consortium data were accessed for GWAS lung cancer summary (11,348 lung cancer cases and 15,861 controls; European ancestry), which includes squamous cell lung carcinomas (LUSC) and lung adenocarcinomas (LUAD). The lung cancer patients were classified according to ICD‐O‐3; SQ: 8070/3, 8071/3, 8072/3, 8074/3; AD: 8140/3, 8250/3, 8260/3, 8310/3, 8480/3, 8560/3, 8251/3, 8490/3, 8570/3, 8574/3; with tumors with overlapping histologies classified as mixed. We also performed sub‐type analyses. Lung cancer was subcategorized as LUSC (3275 cases and 15,038 controls; European ancestry) and LUAD (3442 cases and 14,894 controls; European ancestry).

### 2.2 Instrumental variables

SNPs associated with GERD required *p* < 5 × 10^−8^, linkage disequilibrium (LD, *R* ^2^ ≤ 0.001), met the Hardy–Weinberg equilibrium (H–W) and genetic distance <10,000 kb. Subsequently, major alleles, allele frequencies, β‐values, *p*‐values, and standard errors (SEs) for each SNP were collected. Previous MR studies proved that the application of instrumental variables with high strength can effectively improve the accuracy and efficacy of model estimation. To avoid bias caused by weak proxies, the F‐statistic was calculated and there was no instrumental variable with F‐statistic <10.^23^

After excluding 9 SNPs that strongly associated with the outcome (rs329122, rs2838771, rs6711584, rs2782641, rs773109, rs11762636, rs4382592, rs9615905, rs9529055), 71 SNPs strongly associated with exposure but not with the outcome were obtained. Subsequently, SNPs (rs2145318, rs942065, rs2358016, and rs957345) were removed from the SNPs because they had moderate allele frequencies of the palindromes. We looked for 67 single nucleotide polymorphisms in Phenoscanner (a database of genetic variants holding over 65 billion associations and over 150 million unique results from large‐scale global warming studies;) to assess whether these single nucleotide polymorphisms were associated with genome‐wide significance levels that could affect our results (*p* < 5.0 × 10–8) for other traits. We did not find SNPs strongly associated with the identified carcinogenic factors. A total of 67 SNPs were included in the final MR analysis (Table S1).

### 2.3 MR statistical methods

As instrumental variables, 67 SNPs were included after coordinating the effect alleles of GWAS in GERD and lung cancer. Inverse variance weighted (IVW), weighted median (WM), and MR‐Egger regression were used to analyze the causal relationship between GERD and lung cancer in order to enhance the reliability of causal result.^24^, ^25^ MR analysis included per‐SNP effects incorporated using IVW with slope estimates as slopes of weighted regressions of SNP‐outcome effects on SNP‐exposure effects with zero intercept.^26^, ^27^ Also most specifically, IVW assumes that all the genetic variants are valid. In general, the statistical power of the IVW is dramatically higher than the other two methods. Therefore, IVW was performed as the primary method in our study to scan preliminary associations of GERD with lung cancer. Complementary to IVW, which the WM method presumes that at least half of the instrumental variables are available, the weighted median method calculates a weighted median of estimates of causal relationships between SNPs.^28^ MR‐Egger regression was used to determine whether there was unbalanced pleiotropy and whether exposure was causally responsible for the outcome. When all instrumental variables were null, the MR‐Egger regression method provided consistent estimates, accounting for pleiotropy.^28^ When all the instrumental variables are invalid, the method of MR‐Egger regression offers consistent estimates accounting for pleiotropy.^26^ If the estimates direction of above MR methods were similar, it indicated that the causal effect of GERD on lung cancer was stable and reliable.^24^, ^26^

In the case that the directions of three MR estimates were inconsistent, we tightened the p thresholds of the SNPs associated with GERD from 5 × 10^−8^ to 5 × 10^−9^ and then repeated the MR estimates, which had been described by the study of Chen et al.^29^, ^30^ The odds ratio (OR) and 95% confidence interval (95% CI) were used to display results, representing a risk for lung cancer in GERD patients compared with non‐GERD cases.

### 2.4 Sensitivity analysis

The P‐value of the Cochran's Q test was used in this study to assess the presence of heterogeneity in the analysis, and it was considered that there was no heterogeneity in the causal analysis when Cochran Q‐derived *p* ≥ 0.05. The funnel plot was also used to detect the heterogeneity, and a symmetry plot indicated the absence of heterogeneity.

A fundamental tenet of MR analysis is that instrumental factors may only influence result through exposure; if instrumental variables did not directly affect outcome through altering exposure, the MR assumption would be violated. Thus, the causal relationship between exposure and outcome should be tested for genetic pleiotropy. The bias brought on by genetic pleiotropy may be assessed using MR‐Egger regression analysis, and the pleiotropy's amplitude can be determined using the regression intercept. In addition, to assess the effect of each SNP, the combined effect of each remaining SNP was calculated using the leave‐one‐out method.

## 3 RESULT

### 3.1 MR analysis

As shown as Table 1, the IVW analysis exhibited an increased risk of lung cancer in GERD patients (OR = 1.37; 95% CI 1.16–1.63, *p* = 0. 0003). Meanwhile, the estimates from the weighted median (OR = 1.33; 95% CI 1.08–1.64, *p* = 0. 007) and MR‐egger regression (OR = 1.48, 95% CI 0.57–3.83, *p* = 0.42) analyses showed a consistent direction of the IVW estimates, though the MR‐egger estimate is insignificant.^24^ In addition, using IVW analysis, subgroup analyses also observed similar causal relationships between GERD and LUSC (OR = 1.56; 95% CI 1.26–1.93, *p* = 5.35 × 10^−5^). For lung adenocarcinoma, the MR estimates from the three models were inconsistent (IVW OR = 1.27, 95% CI 1.01–1.60; WM OR = 1.27, 95% CI 0.93–1.70; Egger OR = 0.61, 95% CI 0.18–2.10). We then tightened the SNP‐GERD p threshold to 5 × 10^−9^ and repeated MR estimation, and yielded consistent results from the three MR models **(**Table 1 **)**.

**TABLE 1:** MR estimates for GERD on lung cancer, squamous cell lung cancer and lung adenocarcinoma

Outcome	IVW		WM		MR‐Egger	
	OR (95% CI)	*p*	OR (95% CI)	*p*	OR (95% CI)	*p*
Lung cancer	1.37 (1.16–1.63)	0.0003	1.33 (1.08–1.64)	0.007	1.48 (0.57–3.83)	0.420
Squamous cell lung cancer	1.56 (1.26–1.93)	5.35 × 10^−5^	1.23 (0.92–1.65)	0.159	1.34 (0.41–4.41)	0.634
Lung adenocarcinoma	1.27 (1.01–1.60)	0.039	1.27 (0.93–1.70)	0.109	0.61 (0.17–2.10)	0.440
Lung adenocarcinoma^a^	1.45 (1.09–1.93)	0.011	1.51 (1.04–2.19)	0.032	1.06 (0.20–5.66)	0.949

Abbreviations: IVW, inverse variance weighted; WM, weighted median.

^a^ Using SNPs with a tightened *p* < 5 × 10^−9^.

### 3.2 Sensitivity analyses

Subsequently, sensitivity analyses were conducted to assess the robustness of the primary results, including the Cochran Q test for heterogeneity and MR‐Egger regression for pleiotropy (Table 2). Although heterogeneity was observed in the Q test analysis between GERD and lung cancer (*Q* = 122.70, *p* = 0.0005), the heterogeneity between GERD and LUSC (*Q* = 82.20, *p* = 0.29) and LUAD (*Q* = 49.77, *p* = 0.16) wasn't existed in our outcomes. Since there were no statistically significant intercepts, the results of MR‐Egger regression indicated that pleiotropy appears to be minimal (Table 2).

**TABLE 2:** Sensitivity analysis of the causal association between GERD and the risk of lung cancer

Outcome	Heterogeneity		Pleiotropy	
	Cochran Q	*p*	Intercept	*p*
Lung cancer	122.70	0.0005	−0.003	0.87
Squamous cell lung cancer	82.20	0.29	0.005	0.80
Lung adenocarcinoma	49.77	0.16	0.011	0.71

**FIGURE 1:**
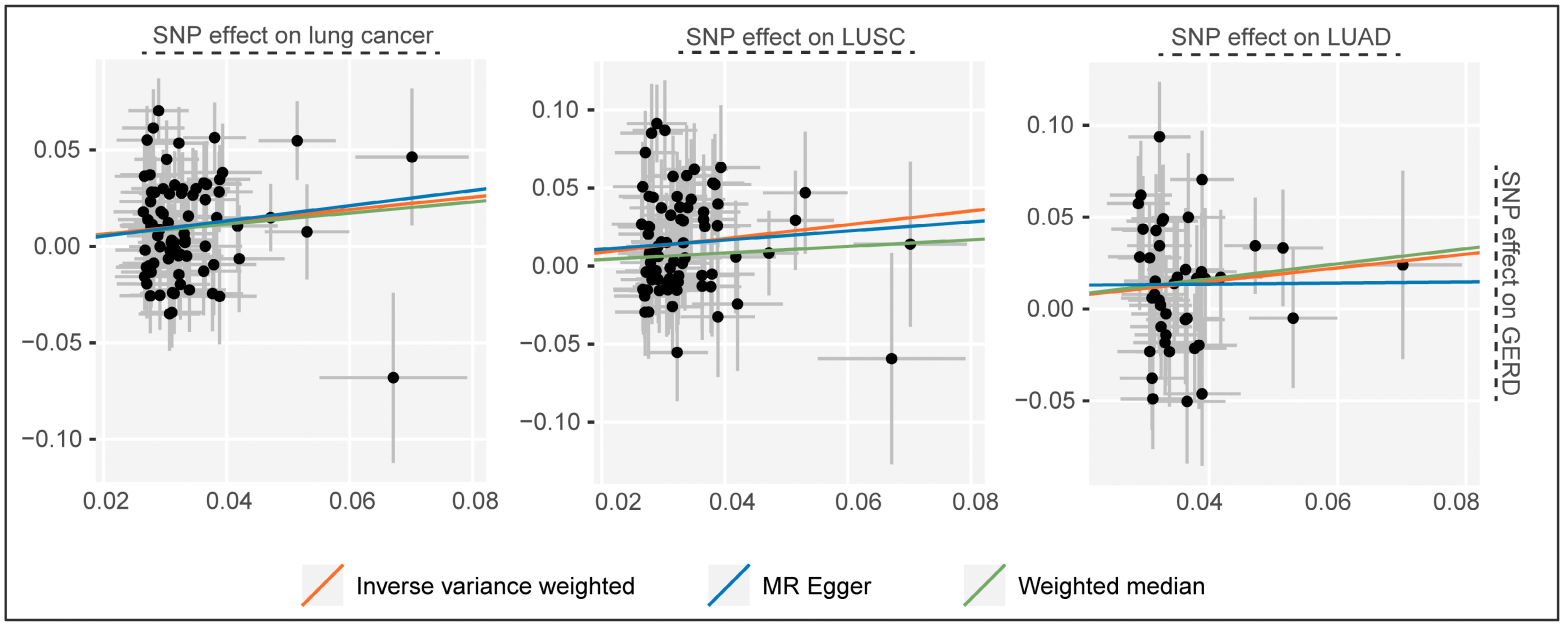
Scatter plots of SNPs associated with GERD and lung cancer, squamous cell lung cancer (LUSC) and lung adenocarcinoma (LUAD).

**FIGURE 2:**
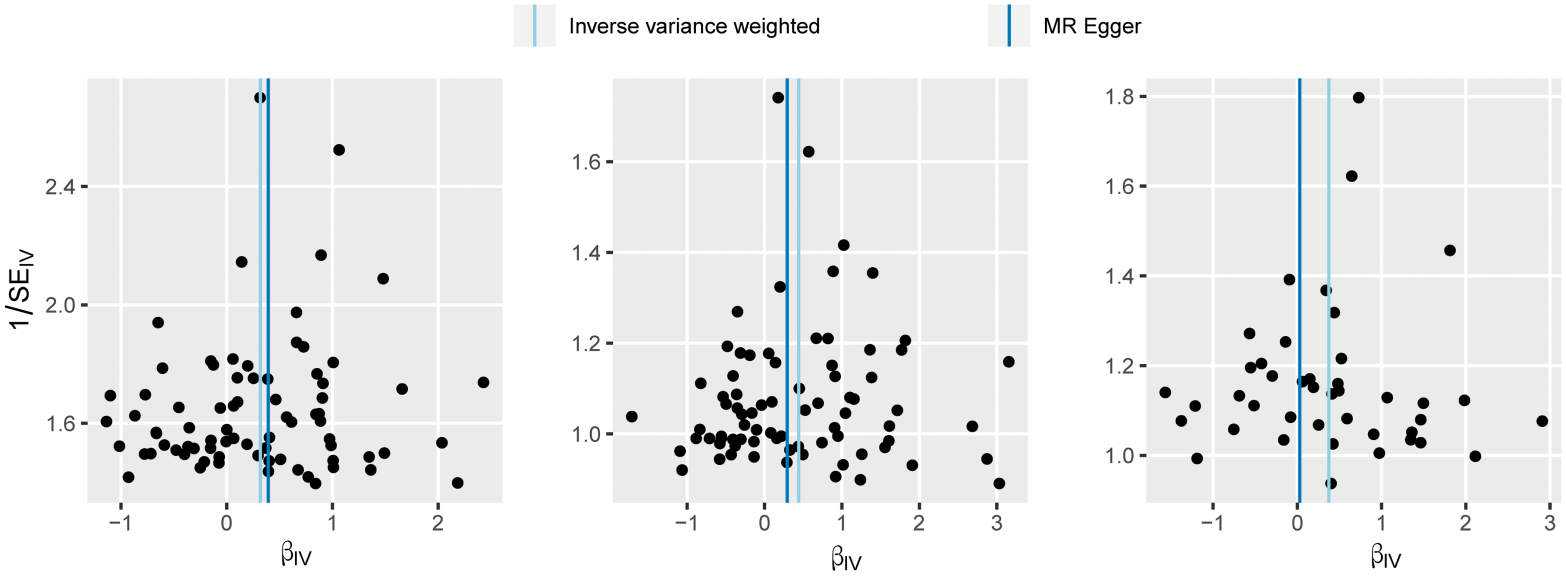
Funnel plots from genetically predicted GERD on lung cancer, squamous cell lung cancer and lung adenocarcinoma.

Additionally, Egger‐intercept for MR‐Egger was not statistically significantly different from 0, indicating that the presence of heterogeneity did not induce any pleiotropic bias into MR estimations **(**Figure 1 **)**. Once an individual SNP was utilized as IV, the funnel plot displayed a symmetric distribution of dots indicating causative interactions, demonstrating that there was less chance that possible bias would have an impact on the causal connection **(**Figure 2 **)**. The “Leave‐one‐out” sensitivity analysis suggested that the results of the IVW analysis of the remaining SNPs were similar to those of the analysis with all SNPs included, and no SNPs were introduced to have a significant effect on the causal association estimates after eliminating each SNP in turn for lung cancer and LUSC (Figure S1). However, for lung adenocarcinoma, removing some SNPs would lead to a consistent but insignificant result, indicating that the causal effect might be violated by some SNPs and conclusion should be cautious.

## 4 CONCLUSION

Using large‐scale GWAS data, this MR analysis revealed that genetically predicted GERD was causally linked to an elevated risk of lung cancer. Specifically, subgroup MR analyses also indicated that GERD was related with the increased risks of LUSC and LUAD. As the first large‐scale MR investigation to examine the link between GERD and lung cancer, it is less susceptible to bias and inverse causation, which may help us better understand the GERD patients' possible risk factors for lung cancer.

The Montreal definition of GERD defines the condition as one in which stomach contents are reabsorbed into the lungs, resulting in troublesome symptoms.^31^ The link between GERD and an elevated chance of esophageal cancer is widely acknowledged, which manifests as esophageal adenocarcinoma after a sequence of metaplasia, dysplasia, and carcinoma.^32^ Furthermore, growing evidence showing that gastroduodenal contents may have an impact on adjacent organs proximal to the esophagus as well as distant systems, especially considering that they lack the similar natural protective systems as the esophagus.^11^ Meanwhile, GERD has been associated with an increased risk of lung cancer in previous studies.^12^, ^13^, ^14^, ^15^

For instance, Vereczkei et al. point out that the prevalence of GERD in patients with non‐small cell lung cancer (NSCLC) is significantly higher than in the common populace, regardless of lung cancer type.^14^ Besides, a population‐based cohort conducted in Taiwan, which include 42,555 individuals, also reveal that GERD patients have a considerably greater prevalence of lung cancer than healthy controls [hazard ratio (RR) = 1.53; 95% CI 1.19–1.98].^12^ Furthermore, GERD has a significant association with lung cancer according to a meta‐analysis that pools three GERD cohorts (pooled RR = 1.47; 95% CI 1.13–1.91).^13^ Overall, these results from observational studies collectively indicate lung cancer risk may be increased by GERD.

However, the available observational studies have been limited by small study size, lack of detailed information on important confounders, such as smoking, related disorders, duration of GERD, and treatment history.^12^, ^18^ Thus, in order to provide constructive suggestions for preventive intervention strategies, it is urgently required to investigate whether there is a causative connection between GERD and lung cancer.

The MR method measures causality by linking an “exposure” and an “outcome,” and reduces the risk of confounding from traditional observational studies, which are commonly used to infer causality, but are time consuming and occasionally impracticable. Noteworthy, the two primary subtypes of NSCLC (LUSC and LUAD), the most prevalent and lethal kind of lung cancer with a high death rate (representing 80–85% of all cases).^33^ Therefore, this study conducted a two‐sample MR analysis and first pointed out a causal relationship from GERD to lung cancer, LUSC, and LUAD. The results of our study indicated that starting treatment as early as possible after the diagnosis of GERD was essential to provide the best clinical outcome and avoid possible complication, such as lung cancer. Additionally, early screening for the risk of lung cancer in GERD patients should be recommended, which may be beneficial in allowing more lung cancer patients earlier diagnosis and curative treatment. Overall, this systematic MR investigation first examine the link between GERD and lung cancer in a large population of European ancestry, providing some lessons for preventive care policies for lung cancer and offering insight on potential points of critical intervention of lung cancer in GERD patients.

Taking all these findings into account, our results confirmed the notion that GERD increased the risk of lung cancer. Several interpretations could account for this significant causality between GERD and lung cancer. Anatomically, the esophagus is surrounded by the trachea and lungs, and multiples studies have indicated that GERD‐induced reflux content can activate inflammatory cascades in susceptible cells of bronchial and lung tissues.^34^ Mechanistically, long‐term chronic inflammation might be involved in tumorigenesis, infiltration, and metastasis by producing inflammatory mediators, participating in angiogenesis and epithelial‐mesenchymal transition.^35^, ^36^, ^37^, ^38^ In addition, results from epidemiologic studies have also yielded that the dominant trend of LUAD in all lung cancer types is similar to the distribution trend of esophageal carcinoma, which might partly support the relation between GERD and LUAD of this study.^39^, ^40^ Therefore, it is worth mentioning that GERD may contribute to the elevated risk of cancer, and the elucidation of the mechanisms between them is extremely important. The findings should be confirmed and potential mechanisms should be explored in future studies, which will enable relevant clinical recommendations to be developed.

Several limitations should be considered when evaluating our study. First, since the biological mechanisms of GERD and lung cancer are not fully elucidated, applying relevant SNPs with unclear mechanisms as instrumental variables has the possibility of violating the core MR hypothesis. Besides, owing to this ethnic heterogeneity, it should be cautious to generalize the results using GWAS data, which are taken from European people and have diverse cultural traditions, to other ethnic groups. Additionally, there is a lack of a formal mediation analysis to explore the possible pathways underlying GERDs association with lung cancer. Last but not least, given the diversity of lung cancer patients, GERD may be causally related to certain lung cancer subtypes, and a more extensive study of lung cancer subgroups may be considered in the future.

In conclusion, this study bolstered the case for a genetic link between GERD susceptibility and lung cancer. Considering the high mortality and morbidity associated with lung cancer patients, it is of significance to recognize and control the risk factor of GERD for lung cancer in order to decrease its prevalence.

## AUTHOR CONTRIBUTIONS

**lin Li:** Conceptualization (lead); formal analysis (lead); methodology (lead); resources (lead); visualization (lead); writing – original draft (lead); writing – review and editing (lead). **Qiaoya Ren:** Formal analysis (lead); investigation (equal); software (lead); validation (equal); visualization (equal). **Qian Zheng:** Formal analysis (equal); validation (equal). **Yuju Bai:** Investigation (equal); project administration (equal); resources (equal); visualization (equal). **Si Si He:** Conceptualization (equal); resources (equal). **Yu Zhang:** Investigation (equal); validation (equal). **Hu Ma:** Conceptualization (lead); data curation (lead); project administration (lead); supervision (lead); writing – review and editing (equal).

## FUNDING INFORMATION

This work was supported by grants from the following sources: (a) Research Programs of Science and Technology Commission Foundation of Guizhou Province (Grant Nos. QKHZC[2020]4Y156); (b) Research Programs of Science and Technology Commission Foundation of Guizhou Province (Grant Nos. QKHJC [2020]1Z062); (c)Guizhou Province Science and Technology Project (Qian Ke He[2019]5406).

## CONFLICT OF INTEREST

All authors declare no conflict of interests.

## COMPLIANCE WITH ETHICS GUIDELINES

Ethics approval UK Biobank received ethics approval from the National Health Service National Research Ethics Service. Written informed consent was obtained for all participants electronically.

## Supporting information


Appendix S1.

Appendix S2.

## Data Availability

Data sharing is not applicable to this article as no new data were created or analyzed in this study.
