# Supplementary material for: Causal associations between gastroesophageal reflux disease and lung cancer risk: A Mendelian randomization study
Source: Cancer Med. 2022 Dec 8;12(6):7552–9. doi: 10.1002/cam4.5498 (PMC10067102; doi:10.1002/cam4.5498)

**Table S1** 67 SNPs were included in the final MR analysis

Figure S1 . Leave-one-out sensitivity analysis for GERD on lung cancer（a）, squamous cell lung cancer （b）and lung adenocarcinoma（c）

**a**


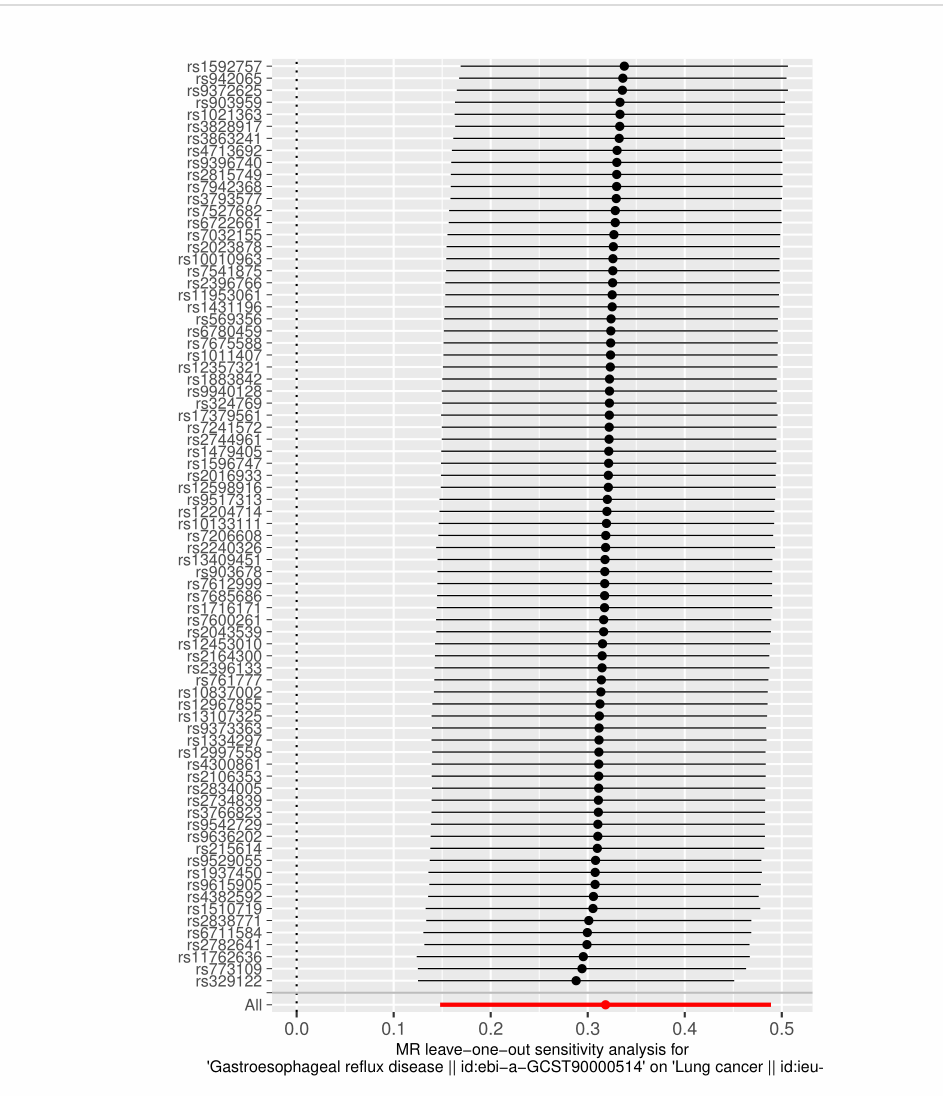


**b**


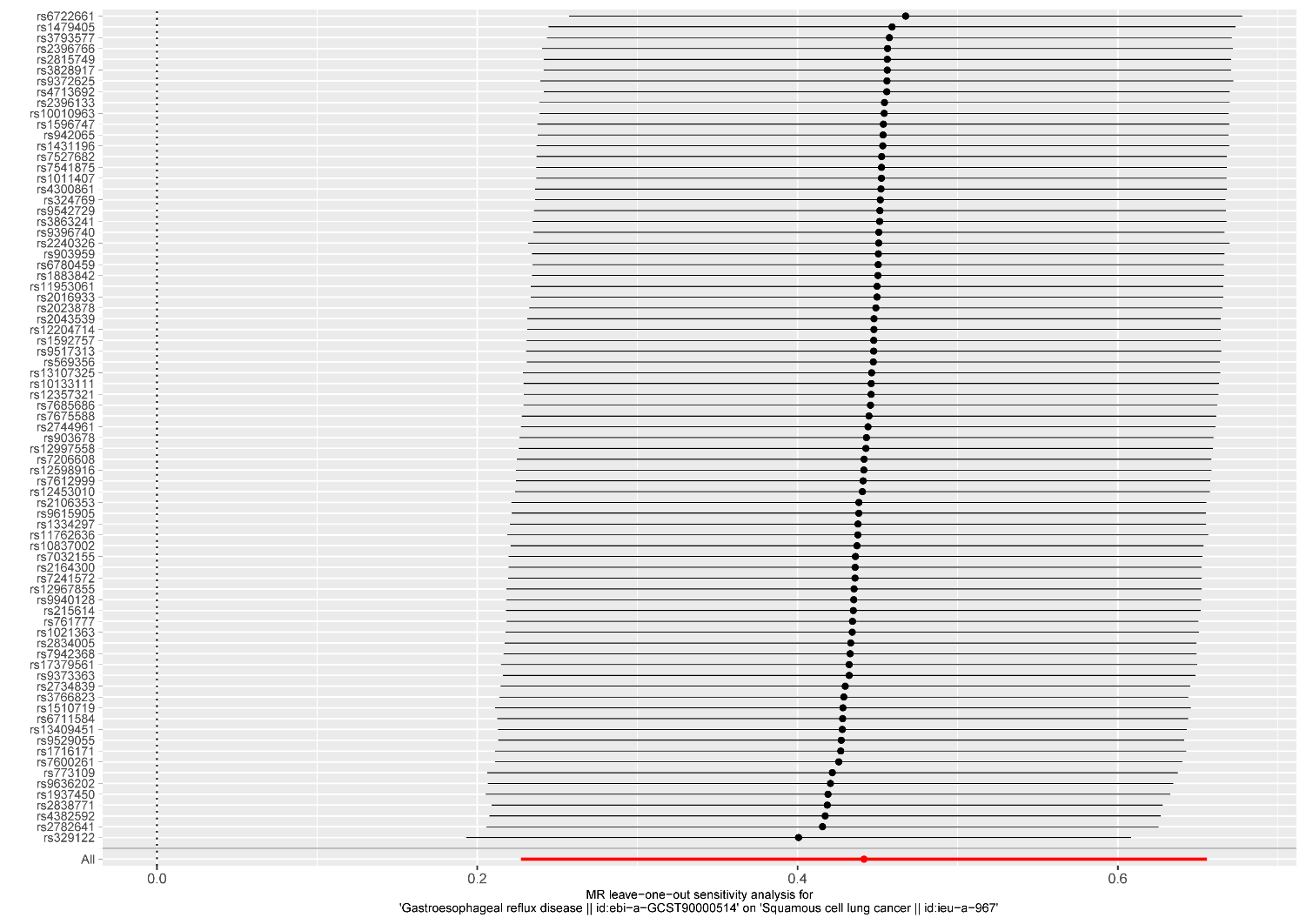


**c**


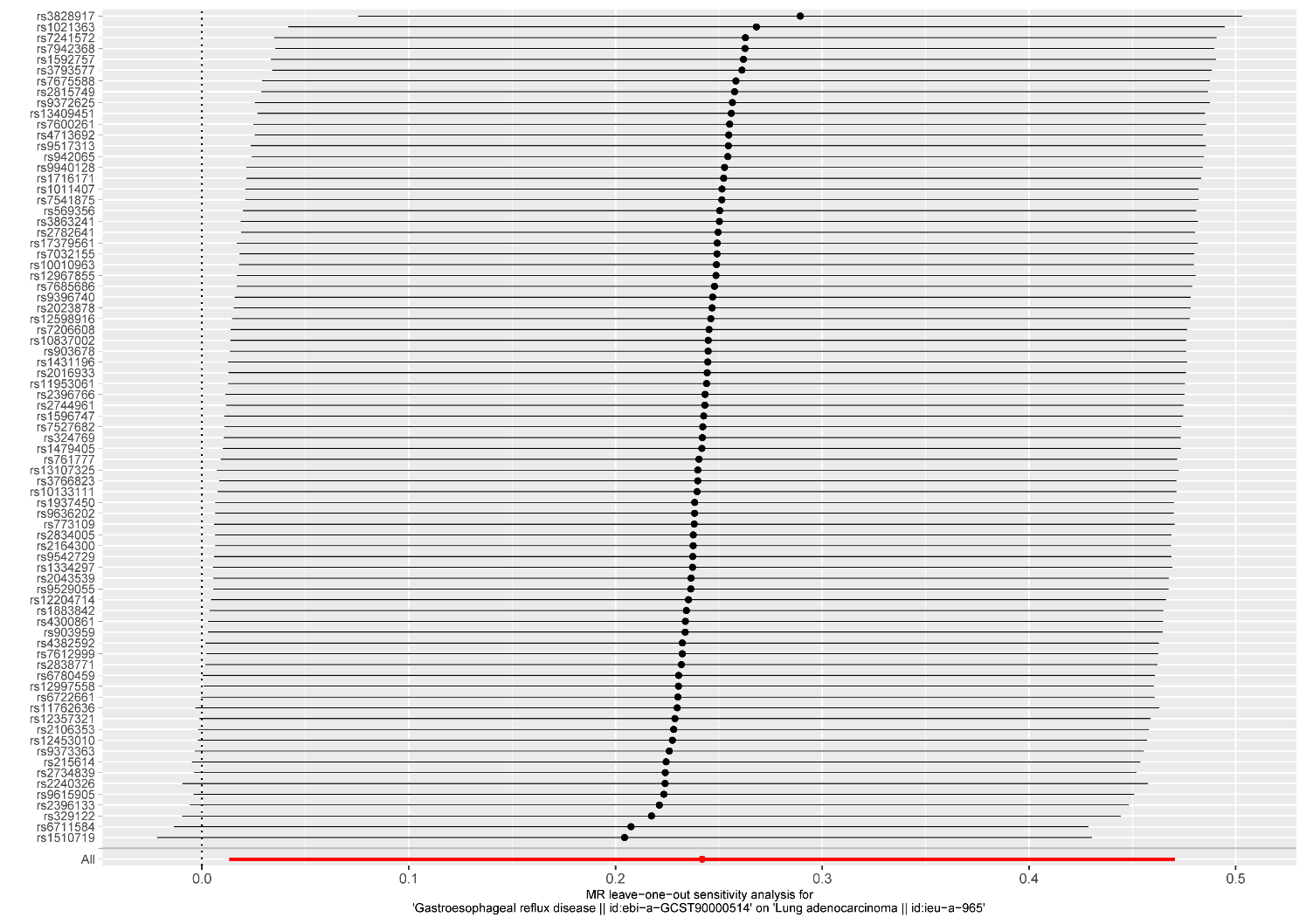

Supplement: Supplementary file 1 — Appendix S1. [file CAM4-12-7552-s001.docx]
